# Costs and benefits of omnivore-mediated plant protection: effects of plant-feeding on *Salix* growth more detrimental than expected

**DOI:** 10.1007/s00442-017-3878-4

**Published:** 2017-05-16

**Authors:** Adriana Puentes, Christer Björkman

**Affiliations:** Department of Ecology, Swedish University of Agricultural Sciences, Box 7044, 750 07 Uppsala, Sweden

**Keywords:** Tri-trophic interactions, Biological control, Natural enemy, Plant growth, Herbivory

## Abstract

Predators can decrease herbivore damage to plants, and this is often assumed to be beneficial to plant growth/reproduction without actual quantification. Moreover, previous studies have been biased towards strict carnivores and neglected the role of omnivorous predators in prey-suppression. Here, we examined the costs (reduction in growth) and benefits (increase in growth) of enemy-mediated plant protection via the omnivorous (prey and plant-feeding) *Orthotylus marginalis*, relative to herbivory by a detrimental insect pest of *Salix* spp. plantations, the beetle *Phratora vulgatissima*. In a first experiment, we compared the cost of adult beetle versus omnivore nymph plant-feeding, and assessed the (non-) additive effects of the two types of damage. In a second experiment, we quantified the reduction in plant damage resulting from beetle-egg feeding by omnivorous nymphs and subsequent benefits to plants. We found that plant-feeding by omnivores negatively affected plant growth and this effect was similar to the cost imposed by beetle herbivory. Furthermore, simultaneous damage effects were additive and more detrimental than individual effects. While egg-predation by omnivore nymphs completely prevented beetle damage to plants, there was no difference in plant growth relative to only herbivore-damaged plants and growth was still reduced compared to control plants. Thus, despite herbivore suppression, there was no benefit to plant growth of omnivore-mediated plant protection and the negative effects of omnivore plant-feeding remained. These results are a first for an omnivorous enemy, and provide novel and timely insights on the underlying assumptions of tri-trophic associations and their use for biocontrol of insect pests.

**Electronic supplementary material:**

The online version of this article (doi:10.1007/s00442-017-3878-4) contains supplementary material, which is available to authorized users.

## Introduction

A greater understanding of the underlying factors regulating interactions between plants, herbivores and enemies of herbivores is currently in great demand (Dicke 2015; Stenberg et al. 2015; Mitchell et al. 2016; Rowen and Kaplan 2016). This is because a remarkable 20–40% of crop losses continue to be attributed to insect pests (Dicke 2015) and there is increased potential for herbivore outbreaks following global environmental changes (e.g. Cornelissen 2011; Aguilar-Fenollosa and Jacas 2014). In addition, the need for reducing insecticide use is an increasing priority and requirement in many places (e.g. EU Sustainable Use Directive 2014). Thus, there is pressing need and timely interest in enhancing and utilising the benefits that natural enemies of herbivores can offer (Dicke 2015; Stenberg et al. 2015; Bruce et al. 2016). Predators can provide protection to plants through direct (consumptive) or indirect (behavioural) effects on herbivores, often reducing damage to plants and increasing biomass (i.e. trophic cascade, sensu Ripple et al. 2016). To determine whether or not enemy-mediated protection is advantageous, knowledge on associated costs (e.g. ecological, physiological) and benefits to plants (e.g. increased growth or reproduction) is required (Dicke and Sabelis 1989; Heil 2008). Yet, seldom are both quantified (Poelman 2015; Dicke 2016); and those few studies that do, are biased towards carnivores and neglect omnivorous predators which have the potential to provide efficient biocontrol of economically important insect pests (e.g. Castañé et al. 2011; Abdala-Roberts et al. 2014). Thus, in this study, we quantified and compared costs and benefits of a plant-herbivore-omnivorous predator interaction to determine whether enemy-mediated plant protection pays off.

Enemy-mediated plant protection will serve in favour of plants when the benefits obtained, in terms of decreased herbivory and subsequent increase in biomass or reproduction, outweigh the costs incurred (Dicke and Sabelis 1989; Karban and Baldwin 1997; Heil 2008; Ågren et al. 2012; Cipollini et al. 2014). Costs can be defined as all negative effects on plants that result from interactions with predators of herbivores, and they can be physiological (e.g. resources taken away from growth), ecological (e.g. detrimental to pollinators), or evolutionary (e.g. constraints on selection) in nature. (Heil and Baldwin 2002; Heil 2008; Kessler and Heil 2011). For example, in the tropical plant *Cordia nodosa*, interactions with ants that act as ‘bodyguards’ increase plant growth when herbivores are present but decrease growth when herbivores are absent, indicating that ant-mediated plant protection is costly (Frederickson et al. 2012). However, with few exceptions, enemy-driven reductions in herbivory have long been assumed to directly translate into positive effects for plants without formal testing (Heil 2008; Poelman 2015; Dicke 2016). Adopting this assumption has resulted in scattered and incomplete knowledge on whether or not it pays off to receive ‘protection’ though natural enemies. While a reduction in herbivory is desirable, without quantification of plant benefits, both the outcome of biocontrol strategies (Stenberg et al. 2015) and understanding the ecology and evolution of plant-herbivore-enemy interactions, will be limited (Kessler and Heil 2011; Dicke 2016).

In particular, assumptions about costs and benefits of plant protection have received even less attention in interactions involving omnivorous enemies, in contrast to those involving ants or parasitoids. Since omnivores feed on both plant- and prey-food, their potential for providing plant protection has been mostly overlooked (Castañé et al. 2011; Dalin et al. 2011; Ågren et al. 2012; Abdala-Roberts et al. 2014; Pérez-Hedo and Urbaneja 2016). Given their plastic feeding habits, interactions with omnivorous predators may be more variable and less intimate (Eubanks and Denno 2000; Kaplan and Thaler 2011; Ågren et al. 2012), thus, thought to be of less ecological relevance for suppressing herbivore populations. However, natural enemies with broad diets are able to survive on other food sources and can be present before herbivore attack (Dalin et al. 2011; Messelink et al. 2015). In addition, like other predators, many can be recruited via plant Volatile Organic Compounds (e.g. Moayeri et al. 2007; Lehrman et al. 2013; Pérez-Hedo and Urbaneja 2015) which could be exploited for artificial attraction (Stenberg et al. 2015), and they can effectively control herbivore populations (e.g. Björkman et al. 2004; Gabarra et al. 2004; Sigsgaard et al. 2006; Oveja et al. 2016). Surprisingly, the effects of plant-feeding by omnivorous predators have been, so far, indirectly assessed from qualitative descriptions of crop lesions, or type of injury based on feeding mode (reviewed by Castañé et al. 2011). This has resulted in the assumption that plant-feeding by omnivorous bugs, with pierce-sucking feeding modes leaving no visible damage marks, has a negligible effect on plants (Albajes and Alomar 2008; Castañé et al. 2011; Dalin et al. 2011; Ågren et al. 2012). Yet, quantitative data to support this assumption is mostly lacking (but see Arnó et al. 2010; Silva et al. 2017 for exceptions); in fact, costs of plant-feeding (e.g. reduction in plant growth) and benefits provided by omnivorous predators (e.g. increase in plant growth) are seldom, if ever, reported. Hence, to determine if omnivorous predators are truly ‘friends or foes’, an estimate of the positive consequences versus the negative effects of plant consumption, relative to the cost of herbivory, is needed.

In this paper, we examined the benefits and costs of enemy-mediated plant protection in different *Salix* spp. genotypes via the predatory bug *Orthotylus marginalis* Reuter (Hemiptera: Miridae), relative to herbivory imposed by the detrimental leaf beetle *Phratora vulgatissima* L. (Coleoptera: Chrysomelidae). *Orthotylus marginalis* is a highly efficient natural enemy of *P. vulgatissima*; it feeds on eggs and early instar larvae (not on adults) of the beetle and has been shown to have a negative effect on the beetle’s population growth rate (Björkman et al. 2004; Dalin et al. 2006). However, there are no actual estimates of the effects of plant-feeding by *O. marginalis* or of the positive effects of reduced herbivory on plants. To our knowledge, this paper presents the first quantification of direct costs of plant consumption by an omnivorous predator, and benefits associated with herbivore reduction. We address the following questions:

1. How does the omnivorous predator *O*. *marginalis* (a) affect plant growth and leaf properties (trichomes, leaf toughness) of *Salix* spp. plants, i.e. what is the cost of omnivore plant-feeding, and (b) how do these responses compare to the effects of adult damage by the herbivore *P. vulgatissima*?
2. When *O*. *marginalis* is present and feeds on eggs and larvae of *P. vulgatissima*, (a) is there a reduction in damage to *Salix* spp. plants and (b) does this correspond to a change in plant growth, i.e. what is the benefit of enemy-mediated plant protection?

We expect damage by *O. marginalis* to be minimal and have an insignificant effect on plant growth or leaf properties of *Salix* spp. plants compared to damage by *P. vulgatissima*, which should be more pronounced and detrimental to plant growth. Prey-feeding by *O. marginalis* should reduce damage and result in a significant increase in plant growth. In other words, we expect benefits of enemy-mediated plant protection to outweigh costs, relative to the negative effects imposed by herbivory.

## Materials and methods

### The plant-herbivore-enemy system

To quantify and compare the costs and benefits of enemy-mediated plant protection relative to herbivory, we examined the interaction between *Salix* (willows) spp. plants, the leaf chewing beetle *Phratora vulgatissima* and the omnivorous bug *Orthotylus marginalis*, referred to often as just *Salix*, herbivore or beetle, and omnivore or mirid, respectively, throughout the manuscript. We chose to focus on *Salix* spp. genotypes (clones) used in Swedish willow plantations since we have substantial knowledge on the levels of herbivore damage and on the effects of *O. marginalis* on the beetle population. In addition, the different genotypes used have been shown to exhibit variation in defence-related traits (physical and chemical properties, e.g. Dalin et al. 2004; Lehrman et al. 2013). Defoliation by *P. vulgatissima*, especially by late-instar larvae, can reduce biomass production by up to 40% and it is the most common herbivore found in plantations (Björkman et al. 2000). In Sweden, *P. vulgatissima* aggregates on preferred host-plants in early May. Until mid- to late-June, females lay several clutches of 5–50 eggs (up to >500 eggs in total, hatching after 15–20 days) on the underside of leaves (Kendall et al. 1996). Larvae pupate in the soil after three instars stages; they overwinter as adults and are univoltine.

In *Salix* plantations, the most abundant natural enemy of *P. vulgatissima* is the omnivorous *Orthotylus marginalis* (Björkman et al. 2003, 2004). It feeds on egg clutches of *P. vulgatissima* and occasionally on early instar larvae (not on adults), and can affect the population dynamics of its prey (Björkman et al. 2004; Dalin et al. 2006). In addition, *Orthotylus marginalis* can plant-feed by pierce-sucking leaf or fruit contents of several deciduous trees including *Salix* spp. (Wheeler 2001). Plant-feeding usually leaves no visible mark; hence, there is no quantification of damage or its effects. However, after a period of repeated plant-feeding, some leaves have been shown to grow with deformations in *Salix cinerea* plants (Liman 2015). *Orthotylus marginalis* has one generation per year and overwinters as eggs (buried under *Salix* bark) with nymphs appearing in mid-May; adults are present until late August.

### Experimental design

To determine the costs and benefits of omnivorous enemy-mediated plant protection compared to costs imposed by leaf beetle herbivory, we conducted two greenhouse experiments at the Swedish University of Agricultural Sciences (SLU), Uppsala, Sweden (59°49′N, 17°40′E). We used 21 different *Salix* spp. genotypes, taxonomically belonging to two species, *Salix viminalis* and *Salix dasyclados* (Table S1). Winter cuttings of these clones had been previously collected from plantations around Uppsala and stored at 4 °C. From storage, cuttings were divided into 15 cm pieces and ten replicates per clone were planted individually in plastic pots (11 × 11 × 12 cm) filled with soil (85% peat, 15% sand). Plants were left to grow for 30 days in a glasshouse (18 °C, 20hL:4hD, RH 50%), fertilised once after 15 days (NPK, 51:10:43+ macronutrients; 1.3 kg m^−3^) and rotated once per week within and between glasshouse benches.

#### First experiment: costs of plant-feeding by predatory bugs

To compare the costs of plant-feeding by *O. marginalis* relative to herbivory, we exposed *Salix* plants to *O. marginalis* (Predators) and to adult *Phratora vulgatissima* (Herbivores). For each *Salix* genotype (*n* = 21), we chose four out of ten replicates originally planted (see above). Each replicate was randomly assigned to one of four treatments (i.e. genotype was not replicated within treatment) in a 2 × 2 factorial design: Control (C) (no herbivores, no predators), Herbivores (H) (two male adult beetles to avoid egg-laying, no predators), Predators (P) (no herbivores, four *O. marginalis* nymphs) and Herbivores and Predators (H + P) (two adult male beetles, four *O. marginalis* nymphs). Note that *O. marginalis* does not feed on adult beetles, hence only costs of plant-feeding can be determined in this first experiment. These treatments allow a comparison of changes in plant growth that occur after adult herbivore damage by *P. vulgatissima* (H treatment) and plant-feeding by *O. marginalis* (no access to prey, P treatment), and an assessment of (non-)additive effects on growth of simultaneous damage (H + P).

Adult beetles were collected from a nearby willow plantation early in the season (first days of May; experiment took place in June) and placed on caged *Salix* plants (a genotype not used in the experiment) in the laboratory under room temperature conditions. Beetles were allowed to feed, mate and freely lay eggs on these plants, which were replaced weekly. *Orthotylus marginalis* nymphs were also collected from willow plantations, 1–2 days before the start of the experiment. This was done by shaking willow branches onto white plastic containers, examining arthropods that ‘crawl out’ and carefully placing in plastic bags those small branch pieces where *O. marginalis* was found. These bags were kept moist in a refrigerator (4 °C) until the start of the experiment; that day, these were emptied onto big plastic containers and mirid nymphs of approximately the same size were chosen. Groups of four mirid nymphs were placed in transparent 30 ml containers (perforated lid) to be released onto the plants (for P and H + P treatments). That same day, pairs of adult male beetles (for H and H + P treatments) were also placed (separately) in these containers following sex determination.

Before releasing the beetles and mirid nymphs according to the experimental treatments, we measured the length of each shoot, and counted the number of shoots and leaves per plant. To determine plant growth during the experiment, the tip of each shoot was marked with a permanent marker (as in Björkman et al. 2008). Each experimental plant (including control plants) was covered with a perforated transparent cellulose bag (hole diameter = 0.5 mm; Baumann Saatzuchtbedarf Co., Germany); bags were loosely placed around the plants but held with a rubber band at the base of the pot, to prevent beetles and mirid nymphs from escaping. We have used these bags in several previous experiments, and they are large enough to allow shoot growth and do not interfere with insect movement. After six days, beetles and mirid nymphs were removed from the plants; we again measured the length of each shoot, and counted the number of shoots and leaves per plant. In addition, to estimate *P. vulgatissima* damage to plants, we counted the number of damaged leaves and of feeding holes per plant (H, H + P treatments).

To examine if plants exposed to herbivore and/or mirid damage exhibited any change in leaf properties, we allowed experimental plants to grow for an additional seven days. After this time, we collected three fully expanded leaves from around the mid-section of each plant to count trichomes, and measure leaf area and toughness. These leaves were first photographed to estimate leaf area using the image analysis software ImageJ (2014, U. S. National Institutes of Health). Then, leaf trichome density was determined as the average number of trichomes crossing a 1 mm line in a stereomicroscope (16×); one measurement was taken on the underside of each leaf, between the mid-vein and the leaf margin. Finally, leaf toughness was measured using a dial tension gauge portable penetrometer (Mitutoyo 546-112, Japan). Units are an index only, expressed as total grams of force required to puncture the thickness of foliage using a 1-g, 0.82-mm pin (Sands and Brancatini 1991).

#### Second experiment: benefits of prey-suppression by predatory bugs

In a separate experiment, we exposed *Salix* plants to mirid nymphs that had access to beetle eggs as prey to determine whether or not harbouring *O. marginalis* provides a benefit to *Salix* plants, relative to the effects of *P. vulgatissima* larval herbivory. Plants were grown as described above, in the same glasshouse and conditions as the first experiment, and for each *Salix* genotype (*n* = 21) we chose four replicates, each to be randomly assigned to one of four treatments: Control (C) (no herbivores, no predators), Herbivores (H) (50 beetle eggs, no predators), Predators (P) (no herbivores, four mirid nymphs) and Herbivores and Predators (H + P) (50 beetle eggs, four mirid nymphs). Beetle eggs were chosen as close to hatching as possible, so that a few days after start of the experiment, larvae would begin feeding on the plants. These treatments allow a comparison of changes in plant growth that occur after larval damage by *P. vulgatissima* and after plant-feeding by *O. marginalis* (no access to prey, P treatment). In contrast to the first experiment that included adult beetles, this experiment allows quantification of benefits conferred by harbouring *O. marginalis* since the H + P treatment allows an assessment of the reduction in damage (and corresponding change in plant growth) that is expected when *O. marginalis* is present and feeds on *P. vulgatissima* eggs/early instar larvae.

We collected *P. vulgatissima* eggs from a nearby willow plantation two days before the start of the experiment. Small branches with several leaves containing eggs were collected from as many different *Salix* plants as possible and kept moist in plastic transparent bags in a refrigerator (4 °C). *Orthotylus marginalis* nymphs were also collected from willow plantations, as described for the first experiment. The day the experiment was due to start, we chose leaves with eggs that were as close to hatching as possible (more yellow in colour, and eyes of larvae almost visible). We counted 25 eggs per leaf and carefully removed any excess eggs under a stereomicroscope (16×) using a pricking needle. Two leaves (25 eggs each × 2 = 50 eggs) were pinned to plants in the Herbivore (H) and Herbivore + Predator (H + P) treatments with two insect needles. Each leaf with eggs was supported by a piece of moistened filter paper and pinned to the main shoot of the plant, in the lower part where *P. vulgatissima* tends to lay most eggs. We made sure that each leaf was in contact or very close to adjacent leaves to facilitate mobility of larvae once they hatched. Plants in the Control (C) and Predator treatment (P) groups were also pinned with two insect needles for consistency. Groups of four mirid nymphs were released onto each bagged plant (for P and P + H treatments) as described for the first experiment. We marked the tip of each shoot with a permanent marker, measured the length of each shoot, and counted the number of shoots and leaves per plant both at the start and end of the experiment (12 days after). In addition, we counted the number of leaves that received herbivore damage and visually estimated a range of damage (lowest–highest) in percentage of area consumed per leaf.

The two experiments were conducted in separate consecutive years for logistical reasons, mostly due to the short time window for insect collection in the field and careful syncing of plant, herbivore and predator availability. Furthermore, given that adult beetles inflict damage at a faster pace than first-instar larvae, the first experiment was conducted for a short-period of time (following Björkman et al. 2008). The number of *P. vulgatissima* beetles and *O. marginalis* nymphs used was based on density estimates from *Salix* plantations, as well as laboratory trials examining predator egg-consumption (Björkman, C.; unpublished data). We used nymphs of *O. marginalis* since their emergence in the spring naturally coincides with the start of egg-laying by *P. vulgatissima*, and they tend to feed more on egg clutches (relevant stage for herbivore control; Björkman et al. 2003) compared to adults. Moreover, the age of experimental plants was based on previous experiments that have examined resistance traits and costs in *Salix* (Dalin et al. 2004; Björkman and Ahrné 2005; Björkman et al. 2008). Lastly, plant growth was measured in terms of cumulative and relative shoot growth, since duration of the two experiments differed. Given the ‘shrub-like’ growing architecture of *Salix* plants, this variable is relevant for management practices (e.g. height growth determines harvest time and resprouting success; Björkman et al. 2000, 2004). From a fitness perspective, it is related to plant vigour (Fritz et al. 2000) and previous studies exposing *Salix* to different types of stress (Zvereva et al. 1997), examining tolerance mechanisms (Hochwender et al. 2013) and effects of mammalian browsing (den Herder et al. 2004), have used shoot length to assess plant responses.

### Statistical analyses

To determine the effects of plant-feeding by *O. marginalis* compared to those imposed by beetle herbivory on plant growth (first experiment), we fitted several linear mixed effects models. All analyses were conducted in R (version 3.1.2, R Development Core Team 2016). We examined plant growth responses in terms of cumulative shoot length (sum of all shoot lengths) and relative growth rate [ln (final length) − ln (initial length)/*t*_(final)_ − *t*_(initial)_]. Separate models were fitted for each growth response (v. 3.1-127 *nlme* package, Pinheiro et al. 2016), but included the same fixed and random factors. Models included as fixed explanatory variables: Herbivores (two levels: 0 and 2 adult male beetles), Predators (two levels: 0 and 4 mirid nymphs) and their interaction; and as a random factor: *Salix* genotypes (*n* = 21). To control for potential differences in size among plants at the start of the experiment we included cumulative initial shoot length (measured before releasing herbivores/predators) as a continuous covariate in these models. In addition, models included a function that estimates individual variances for each treatment combination to account for a non-homogenous error structure. To test the significance of main effects and interactions we used the *Anova* function (which uses Wald tests; *car* package, v.2.1-2, Fox and Weisberg 2011) and of random effects with a likelihood ratio test using the *exactLRT* function (*RLRsim* package, v.3.1-2, Scheipl and Bolker 2015). For contrasts among treatments, we used the *glht* function (*multcomp* package, v. 1.4-6, Hothorn et al. 2016).

To examine if plants exposed to herbivore and/or mirid damage exhibited any change in leaf properties, we again fitted linear mixed effects models. Separate models were fitted for each response variable: trichome density (trichomes mm^−1^), leaf toughness (index, unit-less) and leaf area (mm^2^); trichome density and leaf area were log-transformed to meet assumptions of normality. Models were fitted and significance of factors tested as described above for the growth measurements, except they did not include initial shoot length as a covariate.

To determine whether or not *O. marginalis* provides a benefit to *Salix* plants relative to the effects of larval herbivory (second experiment), we fitted several linear mixed effects models as those described for examining costs. To examine changes in growth, each model included the fixed variables Herbivores (0 and 50 beetle eggs), Predators (0 and 4 mirid nymphs) and their interaction, as well as initial shoot length as a covariate and *Salix* genotypes as a random factor. Main effects, interactions and random factors were tested as described for examining costs. No changes in leaf properties were examined in this second experiment.

## Results

### Costs of plant-feeding by predatory bugs

**Fig. 1:**
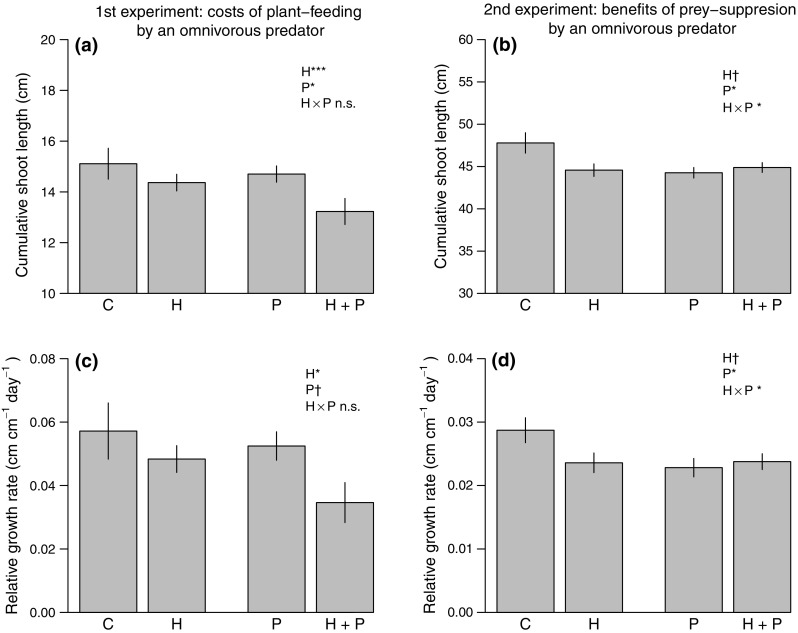
Mean (±SE) cumulative shoot length (cm) and relative growth rate (cm cm^−1^ day^−1^) of *Salix* spp. in experiments examining the costs (first experiment, **a**, **c**) and benefits (second experiment, **b**, **d**) of omnivore-mediated plant protection. Plants were exposed to: no damage (C) (no herbivores, no predators), to damage by the beetle *Phratora vulgatissima* damage (H) (two adult beetles in the first experiment or 50 eggs/larvae in the second experiment, no predators), to plant-feeding by the omnivorous predator *Orthotylus marginalis* (P) (no herbivores, four predator nymphs), and both types of damage simultaneously (first experiment) or predator prey-consumption (second experiment) (H + P) (two adult beetles or 50 eggs/larvae, four predator nymphs). Note: *y-axes* do not start at zero for panels **a** and **c**; summary statistics denote significance of main and interaction effects (*P < 0.05; ***P < 0.001; ^†^ *P* < 0.1)

**Fig. 2:**
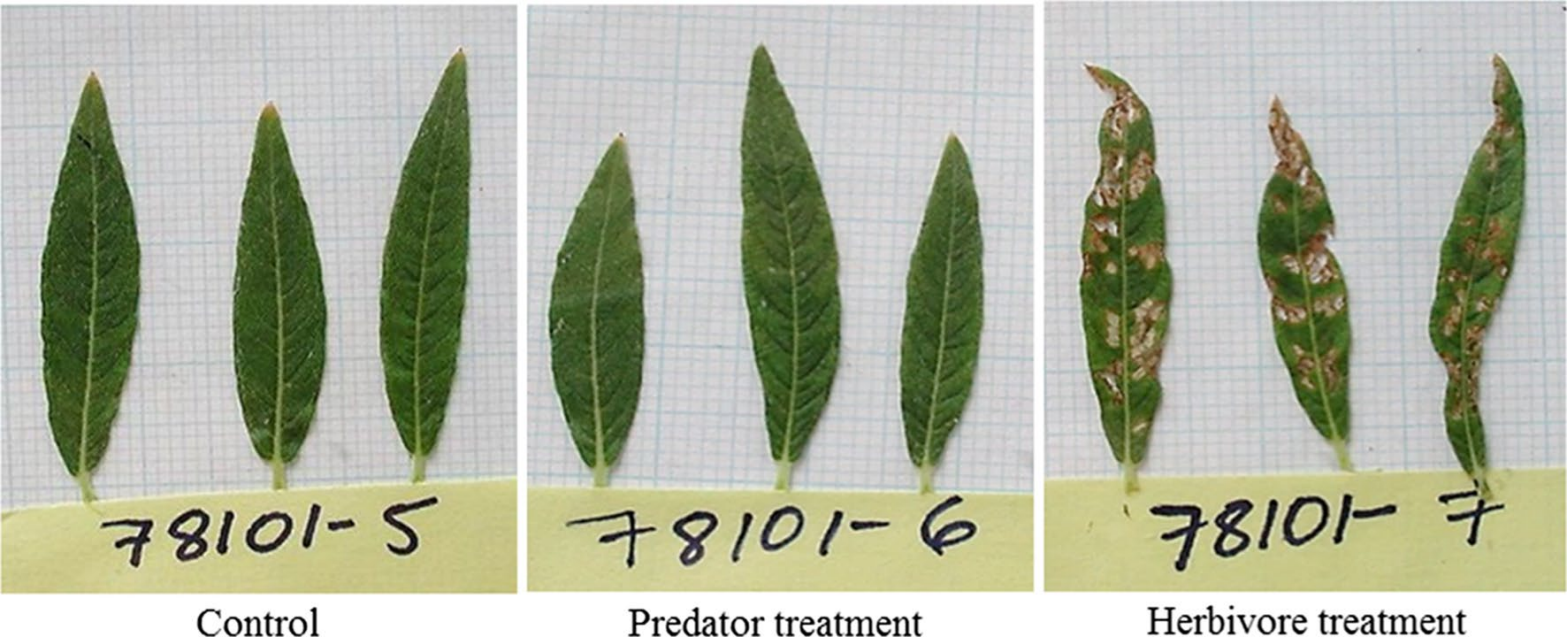
Leaves from *Salix* spp. plants of the same genotype (number 78101) exposed to no damage (Control), only *Orthotylus marginalis* nymphs (Predator treatment), and to adult *Phratora vulgatissima* (Herbivore treatment) after 6 days of feeding. Note the 1-mm graph paper in the background for scale purposes

We found that plant-feeding by *O. marginalis* negatively affected the growth of *Salix* plants and this effect was comparable to that of adult *P. vulgatissima* damage. Plants exposed to mirid nymphs suffered a 5% reduction in absolute growth (16% reduction in relative growth rate, RGR), while plants exposed to adult beetles suffered a 7% reduction (23% reduction in RGR), compared to plants receiving no damage (Fig. 1a, c). There was no visible damage (e.g. leaf deformation, necrosis) to plants in the predator treatment while plants in the herbivore treatment were heavily damaged (Fig. 2). The mean percentage of herbivore-damaged leaves per plant was 64% (standard error, SE, ±2.2) and the average number of feeding holes per leaf was 13.5 (SE, ±0.71). Leaf area damaged by beetles ranged from as low as 1% up to 90–100% for (almost) skeletonised leaves and the presence of predators did not seem to affect the amount of damage inflicted by *P. vulgatissima* (H + P vs. H treatments did not differ; Table S2). The effects of adult herbivores and predators on plant growth were independent of each other (i.e. Herbivore × Predator interaction term not significant; Table 1). This indicates that the treatment effects on plant growth were additive (see Herbivore + Predator bars in Fig. 1a, c). Lastly, growth (cumulative or relative) did not differ significantly among *Salix* spp. genotypes (Table 1).

**Table 1:** Results from mixed models examining the effect of different herbivore (*Phratora vulgatissima*) and omnivorous predator (*Orthotylus marginalis*) damage treatments on plant growth of *Salix* spp. genotypes (*n* = 21), to examine the costs and benefits associated with omnivore-mediated plant protection

Source of variation	1st experiment: costs of omnivore plant-feeding						2nd experiment: benefits of prey-suppression					
	Cumulative shoot length			Relative growth rate			Cumulative shoot length			Relative growth rate		
	*df*	*χ* ^2^	*P*	*df*	*χ* ^2^	*P*	*df*	*χ* ^2^	*P*	*df*	*χ* ^2^	*P*
Fixed effects												
Initial shoot length	1, 59	742.25	**0.000**				1, 57	747.31	**0.000**			
Herbivore treatment (H)	1, 59	7.56	**0.006**	1, 60	6.11	**0.013**	1, 57	3.02	0.082†	1, 58	2.63	0.099†
Predator treatment (P)	1, 59	3.91	**0.048**	1, 60	2.92	0.087†	1, 57	4.63	**0.031**	1, 58	4.90	**0.026**
H × P	1, 59	0.81	0.367	1, 60	0.70	0.404	1, 57	6.6	**0.010**	1, 58	5.55	**0.018**
Random effects		*LRT*	*P*		*LRT*	*P*		*LRT*	*P*		*LRT*	*P*
Genotype/clone		1.06	0.133		0.74	0.189		3.1	**0.035**		10.7	**0.000**

Effects on cumulative shoot length (sum of all shoot lengths, cm) and relative growth rate (cm cm^−1^ day^−^1) were examined in two separate experiments. In the first experiment, a comparison of herbivory vs. omnivore plant-feeding costs was conducted (two levels in each treatment H and P: 0 or 2 adult beetles, 0 or 4 predator nymphs). In a second experiment, the benefits of herbivore egg/larval predation by the omnivore were examined (two levels in each treatment H and P: 0 or 50 eggs/larvae, 0 or 4 predator nymphs). Initial shoot length (start of experiment) was included as a covariate

Significance of fixed and random terms was examined using Wald and likelihood ratio tests (LRT) respectively; significant effects are in bold (*P* < 0.05) or indicated by ^†^ (*P* < 0.10)

Damage by either *O. marginalis* or *P. vulgatissima* did not significantly affect leaf trichomes or toughness of *Salix* spp. plants (Tables 2, S3); however, plants that received herbivore damage tended to have slightly more trichomes than control plants (Table S3). Moreover, the size and number feeding holes inflicted by *P. vulgatissima* was negatively affected by trichome density and leaf toughness, respectively (Table S4; Figs. S1, S2). In addition, leaf area was negatively affected by *P. vulgatissima*, with plants receiving herbivore damage having smaller leaves on average at the end of the experiment (Tables 2, S3). This suggests that leaf growth was reduced in beetle-damaged plants. Lastly, in contrast to results on plant growth, *Salix* spp. genotypes showed significant variation in leaf toughness, trichomes and leaf area (Table 2), indicating that genotypes differ with respect to these leaf properties. Among-genotype variation in trichome density was also detected in a previous study (Dalin et al. 2004) that included some of the same *Salix* genotypes, but variation in leaf toughness has not been previously examined.

**Table 2:** Results from mixed models examining the effect of different herbivore (*Phratora vulgatissima*) and omnivorous predator (*Orthotylus marginalis*) damage treatments on plant growth of *Salix* spp. genotypes (*n* = 21)

Source of variation	Leaf toughness			Trichome density			Leaf area		
	*df*	*χ* ^2^	*P*	*df*	*χ* ^2^	*P*	*df*	*χ* ^2^	*P*
Fixed effects									
Herbivore treatment (H)	1, 59	0.40	0.525	1, 59	1.19	0.275	1, 59	7.7	**0.006**
Predator treatment (P)	1, 59	0.03	0.860	1, 59	0.24	0.622	1, 59	0.30	0.586
H × P	1, 59	0.47	0.493	1, 59	0.47	0.491	1, 59	0.27	0.606
Random effects		*LRT*	*P*		*LRT*	*P*		*LRT*	*P*
Genotype/clone		58.4	**0.000**		83.6	**0.000**		83.6	**0.000**

Leaf properties were only examined in the first experiment, where a comparison of herbivory vs. omnivore plant-feeding costs was conducted (two levels in each treatment H and P: 0 or 2 adult beetles, 0 or 4 predator nymphs). Means for leaf toughness (unit-less index), trichome density (number of trichomes mm^−1^) and leaf area (mm^2^) can be found in Table S3

Significance of fixed and random terms was examined using Wald and likelihood ratio tests (LRT) respectively; significant effects are in bold (*P* < 0.05) or indicated by ^† ^(*P* < 0.10)

### Benefits of prey-suppression by predatory bugs

We found that plants benefited from harbouring *O. marginalis* in the presence of prey by receiving no herbivore damage at all; nymphs were efficient at consuming *P. vulgatissima* eggs and this yielded a complete reduction in damage with no larvae present on any of the H + P treatments plants. In contrast to the first experiment, the combined effect of herbivores and predators on plant growth was non-additive (i.e. significant Herbivore × Predator interaction term; Table 1). When predators were present, the negative effect imposed by herbivores did not occur (high predation by mirid nymphs); however, the negative effect of plant-feeding by predators remained. Plants in the Herbivore + Predator treatment suffered a 6% reduction in absolute growth (17% reduction in RGR) compared to control plants (Fig. 1b, d), and this reduction was no different than that of plants receiving only herbivore damage (H vs. H + P treatment; *z* value = 0.90, *P* = 0.37). In contrast to the first experiment, plants exposed to only mirid nymphs suffered a slightly greater reduction in growth (P treatment: 8% lower growth, 20% lower RGR compared to control plants) than herbivore-damaged plants (H treatment) (Table 1; Fig. 1b, d). For plants receiving larval damage, the mean percentage of herbivore-damaged leaves per plant was 33% (SE, ±6.0) and leaf area damaged by beetles ranged from as low as 5% up to 90%. Again, plants exposed to mirid nymphs showed no visible signs of damage. In contrast to the first experiment, genotypes showed significant differences in growth rate (Table 1). The second experiment ran for a longer period of time compared to the first experiment, and could have allowed differences among genotypes to be expressed and detected, as well as a slightly stronger effect of predators compared to herbivores (P treatment = 8% vs. H = 5% reduction; *z* value = −2.75, *P* = 0.06).

## Discussion

Our study on the costs and benefits of plant protection in *Salix*, mediated indirectly through the omnivorous *O. marginalis*, showed that the cost of plant-feeding by this predatory bug is comparable to the cost imposed by herbivory. While egg predation by *O. marginalis* completely prevented *P. vulgatissima* damage to plants, there was no difference in plant growth relative to herbivore-damaged plants and growth was still reduced compared to plants receiving no damage. These results are contrary to our expectations and the common assumption that plant-feeding by omnivorous bugs with piercing-sucking feeding modes has negligible effects on plants. Our results suggest that the benefits provided by *O. marginalis* do not outweigh the costs, at least in terms of plant growth and compared to *P. vulgatissima* damage in the present setting.

### Costs of plant-feeding by predatory bugs

In contrast to our own and others' frequent expectation that plant-feeding by zoophytophagous natural enemies should be insignificant compared to the damage and effects imposed by herbivores, our study showed that feeding by the omnivorous *O. marginalis* negatively affected *Salix* growth to a similar extent as its herbivorous prey *P. vulgatissima*. These results are surprising, given the apparent lack of damage on plants exposed to *O. marginalis* compared to the evident damage by adult *P. vulgatissima* (Fig. 2). Prolonged feeding by *O. marginalis* can sometimes cause leaf deformations (Liman 2015), and damage inflicted by other Miridae species (hereafter referred to as Mirids) to vegetable crops can be identified by puncture wounds, blemishes, fruit distortion, and necrotic rings on shoots, leaves and stems for example (Castañé et al. 2011; Silva et al. 2017). Even though we did not observe any of these symptoms, *Salix* plants exposed to *O. marginalis* suffered a 5% reduction in growth comparable to the 7% reduction inflicted by herbivores causing striking leaf damage (Fig. 1a, Predator vs. Herbivore treatment). Our results are similar to those of a previous study in which seemingly “undetectable” damage by an omnivorous predatory mite, revealed as severe leaf punctures using microscopy, resulted in a strong reduction in plant height, weight and leaf cover area (Adar et al. 2015). Thus, absence of obvious damage signs can lead to underestimation of plant-feeding by omnivorous natural enemies and, unless explicitly evaluated, this should not be interpreted as having a null effect on plants.

The effect of plant-feeding by *O. marginalis* suggests that responses to this type of damage were detrimental to growth; resources were likely diminished or diverted away to other functions, and different mechanisms could have mediated this effect. Mirids feed by lacerating and ingesting the contents of leaf mesophyll cells (Wheeler 2001), which are primary sites of photosynthesis. Thus, plant-feeding could have affected plant resource availability by reducing or severely damaging photosynthetically active cells, though likely not to the degree that direct defoliation does. While the extent of phloem and xylem feeding is unknown, their piercing-sucking feeding mode results in physiological and biochemical changes in surrounding tissue that could negatively affect plant growth (Wheeler 2001). In addition, Mirid plant-feeding can trigger the activation of localised plant defences at wounding sites and the production of unique blends of Herbivore-Induced Plant Volatiles (HIPVs) through a different signaling pathway compared to leaf chewing herbivores (Leitner et al. 2005; Rowen and Kaplan 2016). Such activation and production of plant defensive compounds is costly (Cipollini et al. 2014) and can occur, for example, in tomato plants following plant-feeding by other Mirid species (Pappas et al. 2015; Pérez-Hedo et al. 2015). This suggests that defence induction could have also contributed to a reduction in *Salix* growth following plant-feeding by *O. marginalis* in our experiment.

In contrast, apart from defence costs, the negative effects of leaf chewing damage are mostly the result of direct consumption of large areas of plant tissue, and subsequent reduction in photosynthetic capacity and resource availability (e.g. Delaney and Higley 2006). In line with this, *P. vulgatissima*-damaged plants did have significantly smaller leaf areas and reduced shoot growth compared to control plants in the first experiment (Tables 2, S3). Nevertheless, it is worth noting that effect sizes of plant growth reductions did not seem to fully reflect the severity of damage by *P. vulgatissima* adults or larvae (see Fig. 2). Several factors could have affected the extent and consequences of leaf beetle herbivory. For instance, while feeding did not change leaf properties as previous studies have also suggested (Dalin et al. 2004), the size and number of *P. vulgatissima* feeding holes was negatively affected by trichomes and leaf toughness, respectively (Table S4; Figs. S1, S2). In addition, several *Salix* spp. genotypes used in this experiment and others have been shown to maintain above-ground biomass even after defoliation by *P. vulgatissima* (Peacock et al. 2002), and different tolerance mechanisms are known to be involved (Hochwender et al. 2012). Thus, the costs of herbivory by *P. vulgatissima* could have been ameliorated by constitutive plant defence traits (inherent leaf properties), and possibly by tolerance mechanisms after damage.

When herbivore and omnivore plant-feeding occurred together, *Salix* plants suffered an even greater decrease in growth than when each occurred independently, suggesting that the costs were additive (Fig. 1a, c; Herbivore + Predator treatment). This is consistent with the idea that each type of feeding damage triggers separate plant reactions. For instance, responses to chewing vs. cell-content-feeding herbivores differ in terms of signaling pathways that are triggered, damage repair mechanisms and defences that are induced to reduce damage (e.g. Leitner et al. 2005). Thus, it is likely that these individual costs of defence add up when both types of damage occur together. However, given that damage was simultaneous, we are unable to determine if and how activation of plant defences by *O. marginalis* affects *P. vulgatissima* feeding, and vice versa. In addition, the presence of *O. marginalis* could have affected *P. vulgatissima* feeding behaviour, resulting in a different cost of herbivory than when *P. vulgatissima* occurs alone. However, differences in levels of herbivore damage between treatments with or without predators (H vs. H + P treatments; Table S2) were not significant, suggesting little effect on beetle feeding behaviour. Lastly, while leaf trichomes have been shown to not affect the predation efficiency or abundance of *O. marginalis* in the field (Björkman and Ahrné 2005), we lack knowledge on whether or not they might influence plant-feeding. In summary, results from this first experiment suggest that if *O. marginalis* is to provide plant protection to *Salix*, the costs associated with plant-feeding can be similar in magnitude to the direct cost of herbivory and should not be neglected, but these costs are likely to be mediated through different physiological mechanisms or responses based on their differential feeding modes.

### Benefits of prey-suppression by predatory bugs

Harbouring *O. marginalis* in the presence of prey resulted in a complete reduction of *P. vulgatissima* damage for *Salix* plants (i.e. *O. marginalis* consumed all beetle eggs and plants did not receive any larval damage). In line with previous studies describing the behaviour of this natural enemy (Björkman et al. 2003), *O. marginalis* was efficient at finding and consuming *P. vulgatissima* eggs. Unexpectedly, plant growth remained reduced despite a lack of herbivore damage (Fig. 1b, d; Herbivore + Predator treatment). The negative effect on plant growth was likely the result of plant-feeding by *O. marginalis*, and this effect was slightly greater than that imposed by *P. vulgatissima* herbivory (Fig. 1b, d; Predator vs. Herbivore treatment). This suggests that *Salix* plants were buffered from *P. vulgatissima* damage, and thus spared the cost of herbivory; but, even with available prey, the cost of plant-feeding by *O. marginalis* remained.

Comparison of our results with previous studies to determine whether enemy-mediated plant protection often or seldom pays off, is currently limited. Most studies examining plant-predator interactions to understand indirect defence (i.e. attracting predators as a means of defence against herbivores) or for potential use as biocontrol, focus on increased predation and reduced herbivore damage, but benefits to plants are mostly unknown or rarely estimated, likely due to logistical difficulties (Heil 2008; Hare 2011; Cipollini et al. 2014; Poelman 2015; Dicke 2016). Only a few studies have recently shown actual positive effects of reduced herbivore damage by natural enemies, in terms of seed production (Schuman et al. 2012; Gols et al. 2015; Pashalidou et al. 2015), and enhanced plant growth (Crowder et al. 2010; Yamawo et al. 2015). Given the lack of knowledge, determining how often or under what circumstances natural enemies fail to provide benefits to plants, despite reducing herbivore damage, is not straightforward. Nonetheless, we do know that even the most voracious of predators do not always decrease herbivore damage to plants and their value as ‘bodyguards’ can vary with ecological conditions (Dicke 2009; Kaplan 2012; Wilson and Woods 2015) and quality of plant genotypes exposed to (Stephan et al. 2016). In the case of *O. marginalis*, its plant protective role has been assumed from highly efficient egg predation; as most of the plant damage is caused by late-instar beetle larvae, predation on eggs and early-instar larvae represents the most important contribution to pest control (Björkman et al. 2003). It has been described as a sedentary predator (“find and stay”) compared to other more active and mobile enemies of *P. vulgatissima*; *Orthotylus marginalis* tends to search for prey in restricted areas, and stays in the vicinity of the eggs it consumes (Björkman et al. 2003). This type of behaviour indicates that they are less likely to move on the next plant or branch, and might switch to plant-feeding if prey is scare within their limited search area. Therefore, if the extent and consequences of plant-feeding are similar under field conditions (which remains to be tested) to those observed in our experiment, *O. marginalis’* actual contribution to plant protection has likely been overestimated and is substantially less than originally thought.

Determining the actual contribution to plant protection by omnivorous predators and how their contribution varies (with, e.g. plant genotype) is essential if they are to be used as biocontrol agents, and if breeding programs to enhance indirect defence are to be further developed (Stenberg et al. 2015). The preference hierarchy of heteropteran omnivores for plant vs. prey foods can be quite plastic (Eubanks and Denno 2000; Kaplan and Thaler 2011) and dependent on plant quality (e.g. leaf nitrogen; Liman et al. 2017), which is of great concern. Nonetheless, omnivorous enemies have been shown to successfully suppress and control herbivorous pests of different crops (e.g. produce crops; Castañé et al. 2011; Abdala-Roberts et al. 2014; Messelink et al. 2015). To buffer against any potential negative effects of omnivore plant-feeding and to extend the time window of effective biocontrol, even provision of supplemental prey food is sometimes used (Oveja et al. 2016). In other cases, omnivorous predators have been proven beneficial by inducing defences through plant-feeding, which has resulted in attraction of other natural enemies, reduced herbivore performance and even direct repellence of pests (Pappas et al. 2015; Pérez-Hedo et al. 2015). To fully understand and utilise the potential of omnivorous natural enemies like *O. marginalis*, greater consideration of the type of outcome desired (pest control or suppression, increased yield, etc.) and of the factors that enhance or hinder prey consumption, is needed.

Full evaluation of costs/benefits of enemy-mediated plant protection is required in an ecologically relevant setting; here, we focused on determining the positive (or absence of) effects on plant growth mediated through consumption of prey, an estimate which is mostly lacking in previous studies. Our two experiments consistently indicate a greater than expected cost of plant-feeding, albeit under glasshouse conditions and a limited period of time. In addition, we used young *Salix* plants and their vulnerability to herbivore/omnivore damage likely varies with age; for example, resistance to mammalian herbivory in other *Salix* spp. has been shown to decrease with age (Tahvanainen et al. 1985). Despite the abovementioned shortcomings, our study is a novel contribution to the few that have examined the costs and benefits of enemy-mediated plant protection, and to the best of our knowledge it is the first for an omnivorous natural enemy, despite an increasing awareness of their role as important predators. Our results provide pioneering and timely insights on the defensive value of plant-enemy associations, which is of current relevance given growing needs to optimize crops for biocontrol of insect pests (Stenberg et al. 2015; Mitchell et al. 2016; Dicke 2016).

## Electronic supplementary material

Below is the link to the electronic supplementary material.

Supplementary material 1 (PDF 173 kb)
