# Supplementary material for: Costs and benefits of omnivore-mediated plant protection: effects of plant-feeding on Salix growth more detrimental than expected
Source: Oecologia. 2017 May 16;184(2):485–96. doi: 10.1007/s00442-017-3878-4 (PMC5487851; doi:10.1007/s00442-017-3878-4)

**Puentes and Björkman -** Costs and benefits of omnivore-mediated plant protection: Effects of plant-feeding on *Salix* growth more detrimental than expected.

Electronic Supplemental Material (ESM)

**Table S1**. List of names and/or identification numbers in Sweden, the taxonomically closest species and sex of the 21 *Salix* spp. genotypes used in the two experiments examining costs and benefits of omnivore-mediated plant protection.

| Genotype/clone | Species | Sex |
|---|---|---|
| 78003 | *S. viminalis* | F |
| 78021 | *S. viminalis* | F |
| 78090 | *S. viminalis* | F |
| 78101 | *S. viminalis* | M |
| 78112 | *S. viminalis* | F |
| 78118 | *S. viminalis* | M |
| 78120 | *S. viminalis* | F |
| 78183 | *S. viminalis* | F |
| 78195 | *S. viminalis* | F |
| 812518 | *S. viminalis* | F |
| 814001 | *S. viminalis* | F |
| 820367 | *S. viminalis* | M |
| 821754 | *S. viminalis* | M |
| 830203 | *S. viminalis* | M |
| 831905 | *S. viminalis* | M |
| Anki | *S. viminalis* | M |
| Eva | *S. viminalis* | F |
| Jorr | *S. viminalis* | M |
| Loden | *S. dasyclados* | M |
| Marie | *S. viminalis* | M |
| Ulv | *S. viminalis* | M |

**Table S2**. Results from mixed models (only fixed effects shown for simplicity; genotype was included as a random factor) examining the effect of predator presence (*Orthotylus marginalis*) on the extent of feeding by the beetle *Phratora vulgatissima* (first experiment: comparison of costs to *Salix* growth of adult herbivory vs. omnivore plant-feeding). Significance of fixed terms was examined using the *anova* function in R (*stats* package, v. 3.4.0; R Core Team). There were no significant differences in the number or size of feeding holes, proportion of leaves and leaf area damaged between *Salix* plants exposed to only adult beetles (H treatment) and those exposed to adult beetles and predator nymphs (H + P treatment).

| Source of variation | Number of feeding holes | | | Size of feeding holes ($mm^2$) | | | Leaves damaged | | | Leaf area damaged ($mm^2$) | | |
|---|---|---|---|---|---|---|---|---|---|---|---|---|
| | d.f. | *F* | *P* | d.f. | *F* | *P* | d.f. | *F* | *P* | d.f. | *F* | *P* |
| Predator presence (H vs. H + P) | 1, 18 | 0.39 | 0.539 | 1, 19 | 0.83 | 0.372 | 1, 18 | 0.10 | 0.753 | 1, 18 | 0.19 | 0.665 |

**Table S3**. Mean (± S.E.) leaf trichome density, leaf toughness and leaf area per plant for each of the treatments for the first experiment only, which examined costs to *Salix* growth of adult herbivory vs. omnivore plant-feeding. Plants were exposed to: control (C) (no herbivores, no predators), herbivores (H) (two adult *Phratora vulgatissima*, no predators), predators (P) (no herbivores, four *Orthotylus marginalis* nymphs), and herbivores and predators (H + P) (two adult beetles, four predator nymphs). Test of main and interaction effects of treatments on plant growth can be found in Table 2 in the manuscript.

| Treatment | Trichomes/mm | Leaf toughness (index) | Leaf area ($mm^2$) |
|---|---|---|---|
| C | 8.29 ± 0.64 | 62.76 ± 1.27 | 222.60 ± 18.00 |
| H | 8.75 ± 0.67 | 62.73 ± 1.33 | 205.62 ± 20.88 |
| P | 8.58 ± 0.69 | 62.22 ± 1.15 | 223.82 ± 19.38 |
| H + P | 8.73 ± 0.66 | 63.20 ± 1.23 | 190.73 ± 18.83 |

**Table S4**. Results from mixed models examining the effect of leaf properties (trichomes, leaf toughness) on feeding by the beetle *Phratora vulgatissima* (number and size of feeding holes) in the first experiment only, which examined costs to *Salix* growth of adult herbivory vs. omnivore plant-feeding. Only plants that received herbivore damage are included in these analyses (plants in the Herbivore and Herbivore + Predator treatments). The size and number of feeding holes was negatively affected by trichome density and leaf toughness, respectively.

| Source of variation | Number of feeding holes | | | Size of feeding holes ($mm^2$) | | |
|---|---|---|---|---|---|---|
| | d.f. | $\chi^2$ | *P* | d.f. | $\chi^2$ | *P* |
| Fixed effects | | | | | | |
| Trichome density | 1, 17 | 1.17 | 0.278 | 1, 18 | 10.61 | **0.001** |
| Leaf toughness | 1, 17 | 4.44 | **0.035** | 1, 18 | 0.47 | 0.489 |
| Random effects | | *LRT* | *P* | | *LRT* | *P* |
| Genotype/clone | | 0.88 | 0.11 | | 0.26 | 0.21 |

**Figure S1.** Relationship between the size of feeding holes inflicted by the beetle *Phratora vulgatissima* and mean plant trichome density (per mm) in *Salix* plants. Only plants that received herbivore damage (Herbivore and Herbivore + Predator treatments) in the first experiment, which examined costs to *Salix* growth of adult herbivory vs. omnivore plant-feeding, were included.

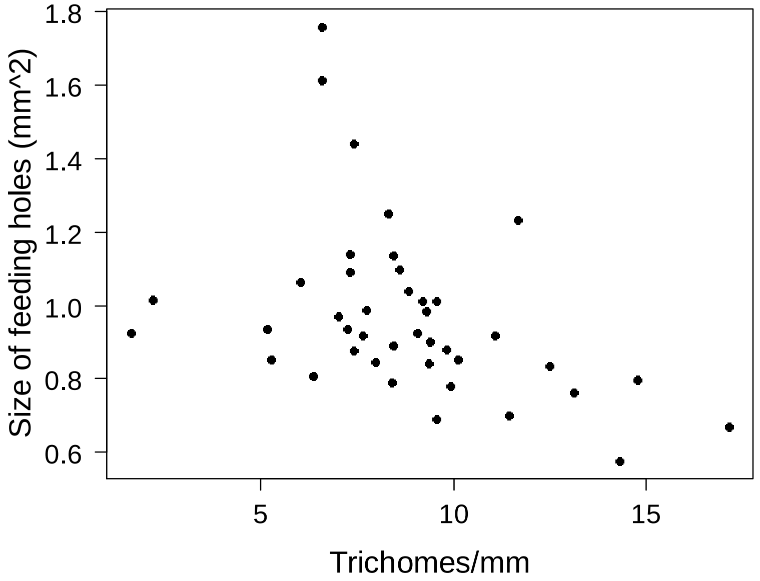

**Figure S2.** Relationship between the number of feeding holes inflicted by the beetle *Phratora vulgatissima* and mean leaf toughness (index) in *Salix* plants. Only plants that received herbivore damage (Herbivore and Herbivore + Predator treatments) in the first experiment, which examined costs to *Salix* growth of adult herbivory vs. omnivore plant-feeding, were included.

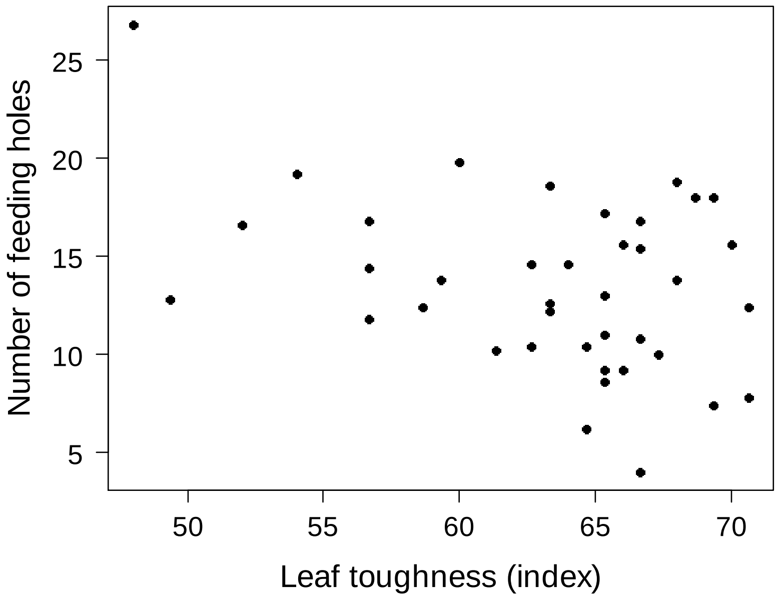

Supplement: Supplementary file 1 — Supplementary material 1 (PDF 173 kb) [file 442_2017_3878_MOESM1_ESM.pdf]
